# The residual laxity of medial collateral ligament after magic point pie crusting MCL released in arthroscopic management of medial meniscus

**DOI:** 10.1016/j.asmart.2024.09.001

**Published:** 2024-09-25

**Authors:** Pinij Srisuwanporn, Suriya Laksawut, Jiradeth Tanulugpairoj, Yottawee Chinakarn, Phichit Khunvejvaidya, Banchong Thantong

**Affiliations:** a Sports Medicine Service, Department of Orthopedics, Rajavithi Hospital, Bangkok, Thailand; b Biomedical Engineering, Department of Medical Innovation, Rajavithi Hospital, Bangkok, Thailand

**Keywords:** Arthroscopic surgery, Knee, Medial collateral ligament, Medial meniscus, Pie-crusting release, Tight knee

## Abstract

**Background:**

In order to do arthroscopic surgery on medial meniscus injuries, there must be enough joint space and good visibility for instrumentation. There is a possibility of iatrogenic cartilage damage if the medial joint space is reduced. Therefore, a medial collateral ligament (MCL) releasing procedure may be necessary for the majority of individuals with medial knee tightness. The MCL residual laxity after pie-crusting release during arthroscopic medial meniscus repair in medial knee tightness were studied in this study.

**Methods:**

Between July 2022 and June 2023, fourteen patients (4 male, 10 female) underwent medial meniscus surgery with pie-crusting release of the superficial MCL. Mean age was 50 ± 10 years (range, 35–63 years). Medial meniscal lesions were meniscus root tear in 10 cases (71.5 %), longitudinal tear in 2 (14.5 %), horizontal tear in 1 (7 %) and radial tear in 1 (7 %). Preoperatively, valgus stress radiographs were obtained. During surgery if arthroscopic exploration revealed medial joint space narrowing after applying valgus force with the knee in 20 degrees of flexion, pie-crusting MCL release was performed. At the 3-month follow-up, valgus stress radiographs were obtained. Residual MCL laxity was assessed by comparing preoperative and 3-month follow-up medial joint space width measurements.

**Result:**

At the 3-month follow-up, no significant increase in the medial joint space width on valgus stress radiograph was observed in comparison to the preoperative. The medial joint space width on valgus stress radiograph was 7.42 ± 1.16 mm preoperatively and 7.47 ± 1.15 mm at 3-month postoperatively (*p* value = 0.914). All patients had no intraoperative iatrogenic cartilage injury and no saphenous nerve injury after operation.

**Conclusions:**

The magic point pie-crusting MCL release is a reliable and useful procedure to arthroscopic surgery in patients with medial meniscal injury and medial knee tightness. Furthermore, percutaneous pie-crusting MCL release had no effect on residual valgus laxity at the last follow-up.

## 1 Introduction

Recently, arthroscopic treatment of knee joint lesions has become popular and widely used by orthopedic surgeons.^1^ Medial meniscus injury is one of the most prevalent lesions of the medial compartment and a cause of knee pain. If left untreated, degenerative changes can occur in the cartilage in the medial compartment of the knee.^2^ However, the knee joint space is often narrow, especially the medial compartment. In particular, the posterior horn of the medial meniscus is one of the most difficult areas to access for knee arthroscopy and the posteromedial compartment is considered to be one of the largest sources of diagnostic error in knee arthroscopy.^3^, ^4^, ^5^, ^6^, ^7^ This is a major problem and it is difficult for surgeons to address medial meniscus pathology during knee arthroscopy. If the medial joint space is narrowed, there is a risk of iatrogenic cartilage injury with arthroscopic instrumentation to treat the medial meniscus. Additionally, attempts to open the medial joint space by applying valgus forces to the knee during surgery may result in rupture of the medial collateral ligament (MCL) or femoral condyle fracture.^8^ In order to solve these problems, recent publications described the local release of the MCL as widening the medial compartment by various methods, including outside-in techniques^9^, ^10^, ^11^ and inside-out techniques.^12^, ^13^, ^14^, ^15^, ^16^, ^17^, ^18^, ^19^, ^20^, ^21^, ^22^, ^23^, ^24^, ^25^

However, a few studies have assessed residual MCL laxity after MCL release, which shown conflicting results. In these studies, the authors proposed MCL release, but did not show specific release structures, locations, or clear anatomical landmarks. Including surgery has been performed by several surgeons and unclear definitions or references of the MCL medial joint space narrowing and adequate medial joint space after MCL release. The aim of this study was to evaluate the residual laxity of the MCL after MCL release for arthroscopic treatment of medial meniscal lesions. By the outside-in percutaneous pie-crusting MCL release was performed at the ‘‘magic point’‘, this technique described by Chernchujit et al.,^25^ it is reliable and very useful in arthroscopic surgery. The hypothesis is that postoperative MCL laxity does not significantly increase following outside-in percutaneous pie-crusting MCL release.

## 2 Materials and methods

### 2.1 Inclusion and exclusion criteria

Inclusion criteria were (1) age >18-year-old, (2) isolated medial meniscus injury, (3) arthroscopic examination showing medial joint space narrowing of the knee, and (4) In medial meniscus surgery, an outside-in percutaneous pie-crust MCL release was performed. Exclusion criteria were (1) associated MCL injury or combined ligamentous injury, (2) associated knee fracture, (3) previous knee surgery, (4) inflammatory arthritis, (5) do not perform a preoperative valgus stress radiograph, and (6) lost to follow-up 3-month after operation. This study was approved by the Hospital Ethics Committee, and informed consent was obtained from all patients.

According to the inclusion and exclusion criteria, 14 patients with medial meniscus injuries from July 2022 to June 2023 were included in this study. All procedures are performed by one surgeon. Age, sex, body mass index (BMI), type of diagnosis, and Kellgren-Lawrence grading^26^ from the standing anteroposterior (AP) knee radiographs were recorded. The medial joint space width of the affected knees was measured according to valgus stress radiographs preoperatively and at 3-month after surgery.

### 2.2 Surgical technique

**Fig. 1:**
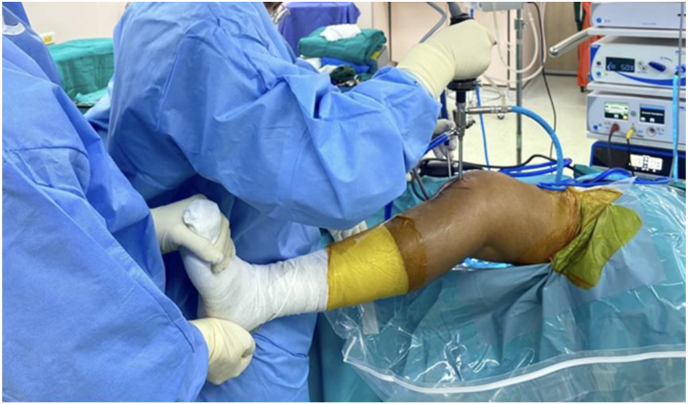
The patient's leg rests on the surgeon's waist and controls valgus force while an assistant externally rotates the leg with the knee is in a 20° flexion position.

Under general or spinal anesthesia, the patient is placed supine on the operating table, and a tourniquet is placed on the proximal thigh. Standard anterolateral (AL) and anteromedial (AM) portals were created, and then arthroscopic examination was performed. In patients with medial meniscal injury, the medial compartment was assessed through the AL portal. The patient's leg rests on the surgeon's waist and applied valgus force while an assistant externally rotates with the knee flexed 20° position (Fig. 1). The medial joint space is evaluated by inserting the probe into the AM portal and using the probe hook (4 mm height; Smith & Nephew plc., London, UK) as a reference. When the probe is inserted through the AM portal, its vertical hooked tip (4 mm height) is placed at the lowest point of the medial femoral condyle at the narrowest medial joint space (Fig. 3c). Percutaneous pie-crusting MCL release is not performed if the medial joint space is above the probe hook level and the entire medial meniscus and posterior root are identified. Meanwhile, percutaneous pie-crusting MCL release is performed if the medial joint space is below probe height and the medial meniscus and posterior root are not well visualized (Fig. 3c).

**Fig. 2:**
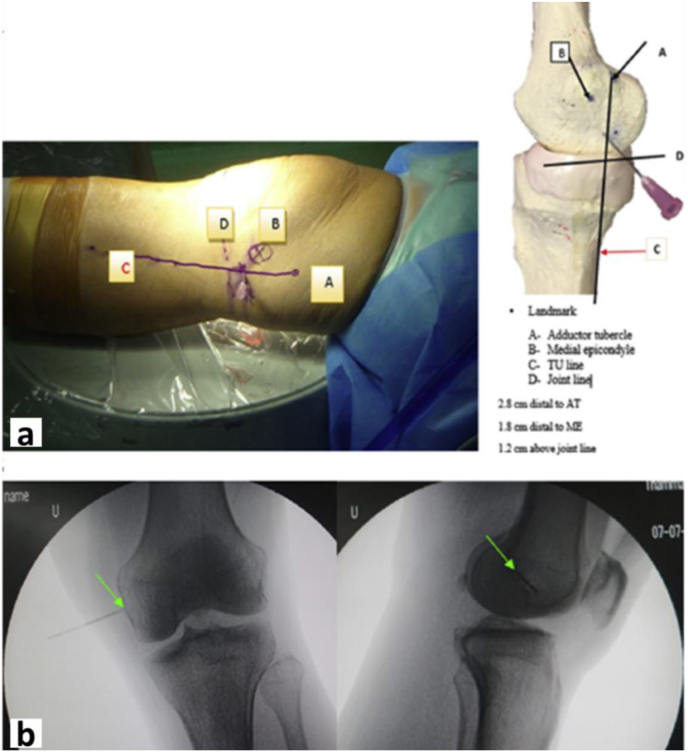
Showing the Outside-in percutaneous pie-crusting MCL release technique by Chernchujit et al.^25^**(a)** The patient is in supine position in full extension with the 18G needle showing the “magic point” of the MCL release on the TU line over the right knee. With external skin marking showing the exact position of the “magic point” in relation with adductor tubercle, joint line and medial epicondyle. The photograh was taken from the left-hand side of the patient. **(b)** The patient is in supine position with right knee in full extension. Fluoroscopic view of the “magic point” in anteroposterior and lateral view of the knee shows the exact site of 18G needle puncture on the medial femoral condyle (TU Thammasat University, Fig. 2 from Chernchujit et al.^25^).

**Fig. 3:**
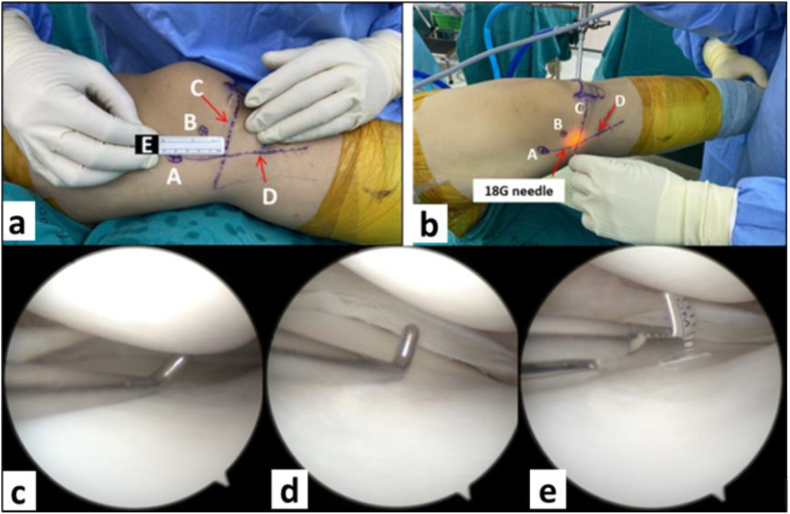
Outside-in percutaneous pie-crusting MCL release. **(a)** The “magic point” is marked 1.2 cm above medial joint line. **(b)** The 18G needle is carefully punctured at the “magic point”. **(c)** The knee in valgus force, external rotation with 20 degrees of flexion, the probe cannot pass vertically through the narrow medial joint space. **(d)** After MCL release the probe is passed vertically through the medial joint space and the field of vision is improved. **(e)** To prevent excessive MCL release, the medial joint space is controlled within 10 mm using a 10 mm plastic ruler. [A = adductor tubercle, B = medial epicondyle, C = medial joint line, D = TU line (Thammasat university line)].

In the outside-in percutaneous pie-crusting MCL release procedure, we used the technique proposed by Chernchujit et al.,^25^ it is reliable, precise and very useful in arthroscopic surgery (Fig. 2a). The technique is performed by identifying anatomical landmarks: (1) adductor tubercle (2) medial epicondyle (3) joint line (4) TU line (Thammasat line: the line extending from the posterior tibial cortex to the adductor tubercle straight line extension) and then draw with a skin marker. On the TU line, a “magic point” was found 2.8 cm distal to the adductor tubercle, 1.8 cm distal to the medial epicondyle, and 1.2 cm above the medial joint line. After confirming the course of the saphenous nerve and vein through transillumination with the arthroscope, the 18G needle was carefully punctured at the “magic point” (Fig. 2, Fig. 3b). When performing the release, the surgeon feels or hears a “crack” on the MCL. After Percutaneous pie-crusting MCL release was performed, the medial joint space is widened and the vertical hook tip can pass through the medial joint space vertically (Fig. 3d). Then use the grasper device to hold a 10 mm plastic ruler insert through the AM portal to measure the medial joint space (Fig. 3e). The medial joint space is controlled within 10 mm to prevent excessive release of the MCL or excessive opening of the medial joint space.

### 2.3 Postoperative management

All patients are advised to wear a long knee brace for 6 weeks to prevent further injury to the MCL and to begin isometric quadriceps strengthening immediately after surgery. Patients undergoing meniscus repair with a posterior meniscal root or radial tears were allowed to bear weight on the toe with a range of motion limited to 0–90°. Gradually gain weight after 6 weeks. The patient was able to return to daily activities 3 months after surgery. Knee flexion beyond 90° should be avoided for at least 4 months, and squatting or jumping should not be performed for at least 6 months. Physical activity allowed 9–12 months after surgery.^27^^,^^28^ Patients undergoing meniscus repair with a vertical longitudinal or horizontal tear, allowing the patient early partial weight bearing and 0–90° range of motion. Exercise greater than 90° and full weight bearing are permitted for 6–8 weeks after surgery. Three months after the operation, the patient returned to daily activities, including stationary cycling and moderate-intensity running. Allow full return to sport after 6–9 months.^29^

### 2.4 Radiographic measurement of the medial joint space width

**Fig. 4:**
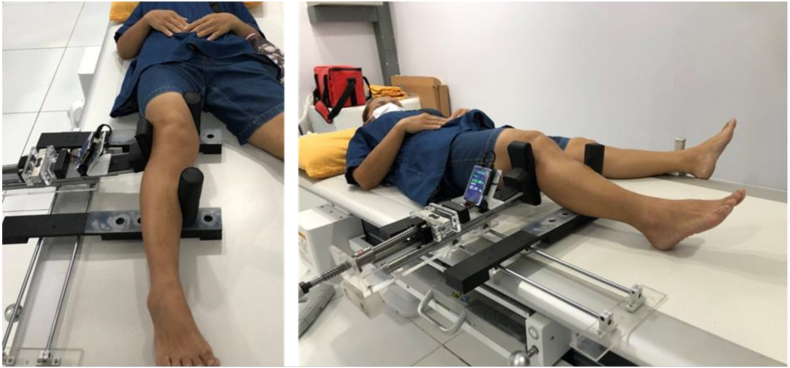
The patient was supine, the knee joint was in a 20° flexion position, the stress device applied a valgus force of 110 N, and an anterior-posterior (AP) knee joint X-ray was taken.

**Fig. 5:**
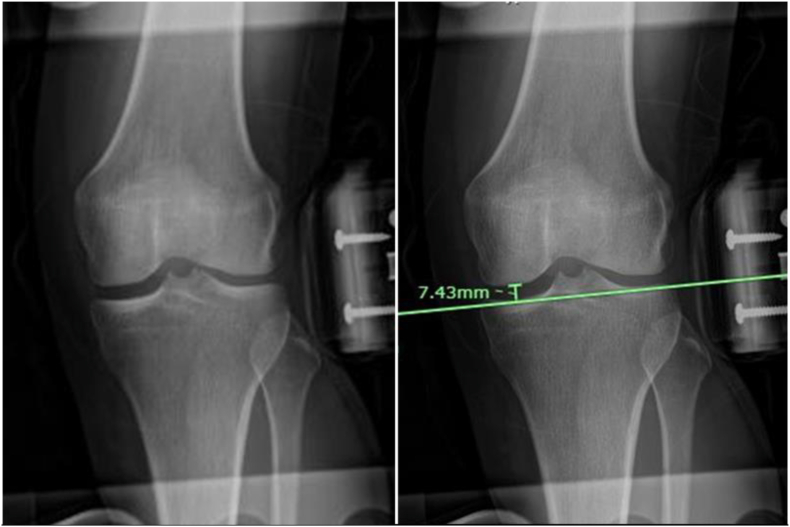
**(a)** Valgus stress radiograph in AP view of left the knee. **(b)** Radiographic measurement of the medial joint space width; drawn line connecting the subchondral bone of the medial and lateral tibial condyles. From this line, draw a vertical perpendicular line to the most distal point of the medial femoral condyle. The distance of this vertical line was recorded as the medial joint space width.

**Fig. 6:**
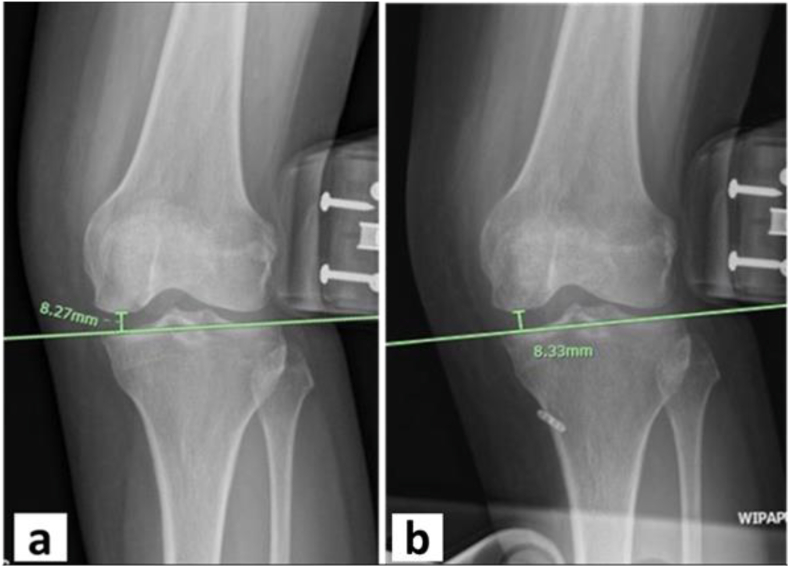
**(a)** Showing the radiographic valgus stress of left knee (AP view) in a patient with a medial meniscus root tear. The preoperative medial joint space width was 8.27 mm. **(b)** Showing the radiographic valgus stress of left knee (AP view) in a patient underwent medial meniscus root repair using the transtibial single tunnel pullout technique at 3 months postoperatively. The medial joint space width was 8.33 mm at 3 months postoperatively.

The stress device (Rajavithi stress radiographic device)^30^, ^31^, ^32^ is applied to the patient's knee. The patient was supine, the knee joint was in a 20° flexion position, the stress device applied a valgus force of 110 N^16,22,24^ (Fig. 4), and an anterior-posterior (AP) knee joint X-ray was taken. The X-ray beam was centered on the joint line, and the tube holder was 1 m away from the cassette. In AP radiograph, drawn a line connecting the subchondral bone of the medial and lateral tibial condyles. From this line, draw a vertical perpendicular line to the most distal point of the medial femoral condyle. The distance of this vertical line was recorded by the PACS software as the medial joint space width in millimeters (Fig. 5, Fig. 6).

### 2.5 Clinical and radiographic evaluation

A valgus stress radiographs were obtained preoperative and at 3-month postoperatively. All of radiographs, the medial joint space width was measured twice at 3-week intervals by two orthopedic surgeons. The severity of collateral ligament injury was also assessed physically exam valgus stress test with the knee flexed 30°; the results were Grade I, ≤5 mm (minor sprains); Grade II, 6–10 mm (partial tears); Grade III, >10 mm (complete tear). The complications of saphenous nerve injury are tested by pinprick sensation and evaluated pain at the medial needle tract of the affected knee.

### 2.6 Statistical analysis

Intra-class correlation coefficients for intra- and inter-observer reliabilities were assessed by using two-way random effects models. ICC and CCC values were assessed as poor when <0.45, moderate to good when 0.45–0.75, and excellent when >0.75.

Due to this study, a small number of patients and repeat the measurement of preoperative and postoperative medial joint space width. Therefore, this study used repeated measures analysis of variance and analysis of longitudinal data to test the significant of differences between preoperative and postoperative medial joint space width. The measurement data are expressed as mean ± standard deviation and P < 0.05 denoted a significant difference. All statistical analyses were performed with STATA 16.0.

## 3 Results

According to the inclusion and exclusion criteria, 14 patients with isolated medial meniscal lesion and medial joint space tightness, who underwent arthroscopic meniscus surgery with percutaneous pie-crusting release of MCL. There are 4 male, 10 female, mean age 50 ± 10 years [range, 35–63 years], mean BMI 26.3 ± 3.8 kg/m^2^ [range, 20.1–33.2]. Medial meniscal lesions were meniscus root tear in 10 cases (71.5 %), longitudinal tear in 2 (14.5 %), horizontal tear in 1 (7 %) and radial tear in 1 (7 %). Kellgren and Lawrence grade 0 in 2 cases (14.5 %), grade I in 3 (21.5 %), grade II in 8 (57 %), grade III in 1 (7 %). Mean operative time 73 ± 11 min [range, 50–85 min] and mean follow-up time 94 ± 3 days [range, 88–98 days] (Table 1). There was no loss to follow-up.

**Table 1:** Patient characteristics.

Patient characteristics (n = 14)	
Age^a^ (year)	50 ± 10
BMI^a^ (kg/m^2^)	26.3 ± 3.8
Gender^b^	
Male	4 (28.5 %)
Female	10 (71.5 %)
Type of diagnosis^b^	
Meniscus root tear	10 (71.5 %)
Longitudinal tear	2 (14.5 %)
Horizontal tear	1 (7.0 %)
Radial tear	1 (7.0 %)
Preoperative Kellgren–Lawrence grading^b^	
0	2 (14.5 %)
1	3 (21.5 %)
2	8 (57.0 %)
3	1 (7.0 %)
4	0 (0 %)
Mean follow-up time^a^ (day)	94 ± 3

BMI body mass index.

a The values are given as the mean and standard deviation.

b The values are given the number of patients, with the percentage in parentheses.

Intra-observer concordance was excellent medial joint space width measurements, with ICC >0.75 for both observers (Table 2). Inter-observer concordances were 0.91 [95 % CI, 0.85–0.96] and 0.92 [95 % CI, 0.87–0.97] for preoperative and postoperative medial joint space width measurements, respectively.

**Table 2:** Intra-observer concordance coeﬃcients for medial joint space width measurement.

Observer	Medial joint space width measurement	
	Preoperative	Postoperative
A	0.95 [95 % CI, 0.90–0.98]	0.96 [95 % CI, 0.92–0.99]
B	0.93 [95 % CI, 0.86–0.96]	0.95 [95 % CI, 0.89–0.98]

Intra-observer concordance coeﬃcients with 95 % conﬁdence intervals.

At the final follow-up, the medial joint space width, as measured from the valgus stress radiographs, was mean 7.42 ± 1.56 mm [range, 5.12–9.05 mm] preoperatively, and 7.47 ± 1.15 mm [range, 5.25–9.08 mm] by 3 months postoperatively. There was no significant difference between the preoperative medial joint space width and that at 3 months postoperatively (p = 0.914) (Table 3).

**Table 3:** Comparison between preoperative and 3 months follow-up Medial joint space width measurement (n = 14).

	Preoperative	Follow-up (3 months)	*p* value
Medial joint space width (mm)	7.42 ± 1.16	7.47 ± 1.15	0.914

There were no intraoperative or postoperative complications, and notably no intraoperative iatrogenic chondral injuries or neurovascular injuries. At 3 months, no patients reported instability and negative valgus stress test with the knee in 30° in all patients compared to the uninjured sides on physical examination. All patients reported slight pain at the medial needle tract of the affected knee for 2 weeks. During the final follow-up, there was no pain on palpation in this area. No cases of hypoesthesia from saphenous nerve injury were found.

## 4 Discussion

Arthroscopic surgical treatment of medial meniscal injuries is one of the most popular arthroscopic procedures. It requires adequate visualization and medial joint space for instrument insertion. Inserting arthroscopic instruments into the medial compartment to treat the medial meniscus may damage the cartilage of the femoral or tibial condyles if the medial joint space is narrowed. Additionally, attempts to open the medial joint space by applying valgus force to the knee during surgery may result in medial collateral ligament rupture or femoral condyle fracture.^8^ To address these issues, recent publications have reported the local release of MCL by various methods to widen the posteromedial compartment space. This technique was first described by Agneskirchner and Lobenhoffer^33^ in 2004, applying the pie crust soft tissue balancing technique to total knee arthroplasty, and more recently several authors have described its use in later arthroscopic procedures. Local release of the MCL to increase the space of the medial compartment by various methods, including inside-out^9^, ^10^, ^11^ techniques and outside-in technique.^12^, ^13^, ^14^, ^15^, ^16^, ^17^, ^18^, ^19^, ^20^, ^21^, ^22^, ^23^, ^24^, ^25^ In addition, there are many arthroscopic tools for MCL release, such as banana blade, electrocautery hook devices, microfracture awls, and needles. Medial knee structure and location depends on surgeon preference, may be deep medial collateral ligament, superficial medial collateral ligament, posterior oblique ligament, or a combination of these. According to several biomechanical studies, superficial MCL is the main stabilizer against valgus force.^34^, ^35^, ^36^, ^37^, ^38^ Biomechanical studies have shown that posterior region of MCL proximal to the joint line has the highest strain when the knee is in extension during valgus loading.^39^^,^^40^ Therefore, this area is thought to be the primary restraint to medial knee opening during valgus force in arthroscopy. Subsequently, Chernchujit et al.^25^ found a “magic point” for percutaneous pie-crusting release of MCL, this technique is reliable, precise and very useful in arthroscopic surgery. The technique is performed by identifying anatomical landmarks: (1) adductor tubercle (2) medial epicondyle (3) joint line (4) TU line (Thammasat line: the line extending from the posterior tibial cortex to the adductor tubercle straight line extension) and then draw with a skin marker. On the TU line, a “magic point” was found 2.8 cm distal to the adductor tubercle, 1.8 cm distal to the medial epicondyle, and 1.2 cm above the medial joint line (Fig. 2a).

Literatures shown that releasing the MCL significantly improved vision and widened the medial joint space. At the same time, problems caused by these techniques may lead to postoperative knee residual MCL laxity or medial knee instability. However, there are a few studies that have assessed the residual MCL relaxation after MCL release. Fakioglu et al.^16^ reported a prospective study of 18 patients who had percutaneous MCL release during arthroscopic medial meniscectomy. Valgus stress radiographs were 7.1 mm (3.7–9.6) preoperatively, 9.1 mm (6.2–11.3), 8.0 mm (5.3–10.1), and 7.2 mm (3.9–9.8) at 1-week, 3-month, and 6-month follow-up, the difference was statistically significant (p < 0.0001). Han et al.^24^ reported a retrospective study of 60 patients, underwent arthroscopic medial meniscal surgery with pie-crusting release on the posteromedial complex of the knee. The radiographic joint space width was 5.97 ± 0.8 mm preoperatively, 9.2 ± 1.1 mm and 6.1 ± 0.9 mm at 1-week and 3-month follow-up, showing no differences between preoperative and 3 months postoperative measurement (p > 0.05). Joen et al.^18^ reported a retrospective study of 814 patients who underwent arthroscopic surgery of the medial compartment of the knee and percutaneous pie-crusting MCL release. The side-to-side difference in valgus gap was not significantly increased compared to the preoperative value in the release group [preoperative - 0.1 ± 1.3 mm; follow-up, −0.1 ± 1.4 mm; (p > 0.05)]. Lons et al.^22^ reported a prospective study of 40 patients who underwent arthroscopic management of isolated medial meniscus lesions and a pie-crusting release at the junction of the posterior two-thirds of the medial compartment. The opening of the tibiofemoral joint space was significantly larger at 6-week on measurements: 1.1 ± 1 mm [−0.6 to 3.2 mm; (p < 0.0001)].

Nonetheless, the results of these studies regarding the residual laxity of the MCL have been contradictory. In most studies, the authors propose that MCL release does not show specific structures, locations, or reliable anatomical landmarks of the medial knee structure or MCL release. As shown in a study by Fakioglu et al.,^16^ following MCL release, MRI findings revealed variable and different locations at the MCL release point. Without a reliable landmark, multiple attempts for MCL release may endanger to surrounding structures including as cartilage, meniscus, saphenous nerve and vein. Among other things, the operation is performed by several surgeons. Some studies were unclear about the definition or referencing of medial joint space narrowing, which is depended on the discretion of the individual surgeon. The definition or reference of adequate medial joint space following MCL release is also unclear and is at the discretion of each surgeon. One of these studies performed radiographic valgus stress test at 1 week in the early postoperative period. This may lead to painful and progressive MCL laxity. Several studies assessed residual laxity in the MCL compared to the contralateral side. Therefore, this study is working on improving these limits to reduce bias and improve the accuracy of the data. This study design is a prospective study with surgery performed by a single surgeon. The location and anatomical landmarks for outside-in percutaneous pie-crusting MCL release by using the ‘‘magic point’‘. This method described by Chernchujit et al.,^25^ it is clearly anatomical landmarks, precise and reliable to use in arthroscopic surgery. Our study defined narrowing of medial joint space by the width of the probe hook. Meanwhile, adequate medial joint space after MCL release by checking that the medial joint space did not exceed 10 mm by using a 10 mm plastic ruler, to prevent excessive releasing of the MCL or excessive opening of the medial joint space after MCL release. Our study waited 3 months to ensure complete healing of the MCL and then perform radiographic valgus stress to prevent early progression of MCL laxity.

The results of our study showed that the patient's knee joint had no objective medial instability at 3 months after operation, and the joint space width of the valgus stress radiograph was 7.42 ± 1.56 mm [range, 5.12–9.05 mm], which was not significantly different compared with preoperative 7.47 ± 1.56 mm [range, 5.25–9.08 mm]. There are four key factors for healing of the MCL and knee stability after the MCL was released in our study: (1) In our technique, using ‘‘magic point’’ for outside-in percutaneous pie-crusting release of MCL. This method described by Chernchujit et al.,^25^ it is reliable, as its anatomical landmarks can be accurately located. (2) In our study, Pie-crusting MCL release is located 1.2 cm above the joint line, and is located at the posterior and proximal portions of the superficial MCL, which includes the deep MCL.^41^ (Fig. 2b). Several studies have shown that proximal MCL injuries have better healing rates than distal and mid-substance MCL injuries, which are observed when pie-crusting MCL release through the joint line. Therefore, when nonoperative management is employed for MCL injury, pie-crusting MCL release 1.2 cm over the joint line is consequently more predictable and successful.^42^^,^^43^ (3) Intraoperative controlled the medial joint space opening after MCL release by verifying that the medial joint space within 10 mm to prevent excessive release of the MCL or excessive opening of the medial joint space. (4) Patients wear a brace for at least 6 weeks after surgery and wait 3 months to ensure complete MCL healing before undergoing valgus stress x-rays.

This study has some limitations. First, the study includes a small sample size, which is a major limit. Second, intraoperative valgus force was applied to the knee joint at the surgeon's waist for evaluate medial knee tightness, which amount of force is determined by the surgeon. However, this study attempted to reduce bias in operations performed by a single surgeon and valgus stress radiographs can be used to predict medial knee tightness before surgery if the medial joint space width is less than 10 mm. Third, this study has short-term follow-up. Therefore, more patients with longer follow-up time needed to be recruited in the future.

A patient with medial meniscal injury with medial knee tightness. The outside-in percutaneous pie-crusting MCL release at the magic point allows easier access to surgical instruments during arthroscopic surgery in these patients without endangering the articular cartilage. Furthermore, by releasing the MCL at the magic point, injury to the meniscus and articular cartilage can be avoided, which these complications can occur when the MCL being released through the joint line. A percutaneous pie-crusting MCL release by using of “magic point” technique^25^ is a safe procedure without concern for iatrogenic residual MCL laxity or valgus laxity, saphenous nerve injury and pain at the medial needle tract of the affected knee.

## 5 Conclusion

The magic point pie-crusting MCL release is a reliable and useful procedure to arthroscopic intervention in patients with medial meniscal injury and medial knee tightness. Furthermore, percutaneous pie-crusting MCL release had no effect on residual valgus laxity at the last follow-up.

## Funding acknowledgements

This study was not supported by any sponsor or funder.

## Declaration of competing interest

No conflict of interest in this study.
